# Transformer‐Based Deep Learning Model for Predicting Recurrence in High‐Grade Glioma

**DOI:** 10.1002/cam4.71740

**Published:** 2026-05-08

**Authors:** Xin Wang, Mingjun Ding, Dan Zong, Pudong Qian, Xia He

**Affiliations:** ^1^ Department of Radiotherapy, The Affiliated Cancer Hospital of Nanjing Medical University Jiangsu Cancer Hospital, Jiangsu Institute of Cancer Research Nanjing China; ^2^ Collaborative Innovation Center for Cancer Personalized Medicine Nanjing Medical University Nanjing China

**Keywords:** deep learning, high‐grade glioma, MRI, recurrence, transformer

## Abstract

**Introduction:**

The first year after treatment for high‐grade glioma (HGG) is recognized as the peak interval for recurrence. Accurate prediction of recurrence during this period is critical for timely management and early intervention. This study aimed to develop a fusion model that integrates MRI‐derived features with clinical variables.

**Methods:**

A retrospective analysis was conducted on 309 postoperative patients with HGG who received intensity‐modulated radiation therapy (IMRT) at Jiangsu Cancer Hospital from 2016 to 2023. Patients were randomly assigned (7:3) to training (*n* = 216) and test (*n* = 93) cohorts. Clinical variables were screened using univariate analyses. Regions of interest (ROIs)—including the postoperative cavity and peritumoral edema—were manually segmented on T2‐weighted images by two senior physicians using ITK‐SNAP. Pretrained DenseNet‐121, DenseNet‐201, and DenseNet‐169 architectures were used to extract deep‐learning features. These features were used to construct deep‐learning models; all models were trained using fivefold cross‐validation on the training cohort. Gradient‐weighted class activation maps (Grad‐CAM) were generated to visualize model attention. Model performance was evaluated using receiver operating characteristic (ROC) analysis, calibration curves, and decision curve analysis (DCA).

**Results:**

Of the 309 patients, 134 experienced recurrence within one year. Integrating clinical variables with transformer‐based models yielded substantial performance gains. Among the integrated models, Combined‐Transformer‐DenseNet121 achieved the best performance, with an AUC of 0.903 (95% CI, 0.862–0.943) in the training cohort and 0.747 (95% CI, 0.642–0.852) in the test cohort. Calibration fidelity was improved; Hosmer‐Lemeshow tests indicated excellent agreement between predicted and observed outcomes (HL statistic, 0.166 [training] vs. 0.158 [test]). DCA demonstrated superior clinical utility in both training and test cohorts, consistently yielding the highest net benefit across threshold probabilities when model‐predicted probabilities were applied.

**Conclusions:**

The proposed model demonstrated superior performance for predicting one‐year recurrence in high‐grade glioma compared with traditional approaches, offering high accuracy and facilitating early identification of high‐risk patients.

## 1 Introduction

Gliomas, the most common tumors of the central nervous system (CNS), are classified as grades 1–4 under the 2021 WHO classification. Grades 3 and 4 are designated high‐grade gliomas (HGGs) [1] and primarily comprise IDH‐wildtype glioblastoma (GBM), IDH‐mutant astrocytoma, and IDH‐mutant oligodendroglioma with 1p/19q codeletion. These malignancies are characterized by high mortality, frequent recurrence, and poor prognosis [2].

Despite comprehensive management, including maximal safe resection followed by the Stupp protocol [3] (radiotherapy with concurrent and adjuvant temozolomide) and newer modalities such as Tumor Treating Fields (TTFields) [4], clinical efficacy remains limited. In clinical trial ChiCTR2100046667, two HGG subgroups receiving postoperative radiotherapy with concurrent and adjuvant temozolomide (TMZ) achieved median progression‐free survival (mPFS) of 10.0 months (95% CI, 3.8–16.2) and 11.0 months (95% CI, 7.1–14.9), respectively [5]. These results indicate that, even with intensive regimens, mPFS generally remains below one year [6]. Notably, the first year after treatment represents both the peak interval for recurrence or progression [7] and the period during which standard adjuvant temozolomide cycles are typically completed. Accordingly, recurrence or progression within one year after treatment serves as a critical prognostic endpoint.

AI in medicine, and particularly in neuro‐oncology, is an emerging field [8]. A recent systematic review and meta‐analysis assessed the diagnostic accuracy of machine‐learning algorithms for forecasting HGG recurrence [9] and highlighted strong performance—particularly for support vector machines (SVM)—in identifying glioma recurrence. Deep learning (DL), as a specialized branch of machine learning [10], employs deep neural architectures, most commonly convolutional neural networks (CNNs) and other multilayer networks, to automatically learn abstract, high‐level representations from data, especially medical images. Multiple studies have shown that DL surpasses conventional ML, especially in analyzing high‐dimensional data and modeling complex feature interactions [10]. Properly developed and validated DL models can therefore be used as reliable instruments for personalized outcome prediction.

Chelliah et al. [11] estimated GBM survival by applying DL to the first post‐radiotherapy brain MRI, achieving reliable accuracy. Most prior studies have used preoperative MRI to extract features for predicting survival in patients with glioma [12, 13]. Because the extent of resection substantially influences HGG prognosis [14], postoperative alterations in tumor biology and the microenvironment may be captured by subvisual imaging biomarkers. MRI obtained between surgery and the initiation of radiotherapy can reflect these changes. Deep‐learning architectures such as MobileNet [15], ShuffleNet [16], DenseNet [17], and ResNet [18] are capable of directly extracting features from raw medical images, capturing comprehensive visual information [19]. These extracted features can serve as valuable prognostic biomarkers that complement the WHO classification in predicting outcomes for patients with HGG.

DL models have shown strong performance in glioma grading, molecular‐subtype prediction, and prognostication. Yu et al. [20] developed a DL framework that improved grading accuracy in HGGs. Mahootiha et al. [21] used a pretrained DL tool to extract MRI‐based features from preoperative T2‐weighted images and combined them with clinical variables to train a model that accurately predicted postoperative event‐free survival in pediatric low‐grade gliomas (pLGGs). Niu et al. [22] developed MRI‐based transformer models using the cross‐scale attention Vision Transformer (CrossFormer) and a complementary radiomics model to identify gliomas that are IDH‐wildtype with TERT‐promoter mutations.

Traditional predictive models are commonly constructed from readily available clinical features, which remain the cornerstone of prognostic modeling. Bianconi et al. [23] evaluated clinical variables associated with the decision to pursue surgery to identify general and disease‐specific preoperative predictors of 12‐month mortality in older patients (≥ 75 years) newly diagnosed with HGG. Systemic inflammatory markers also provide predictive value. A retrospective study [24] used neutrophil‐to‐lymphocyte ratio (NLR), systemic immune‐inflammation index (SII), and systemic inflammation response index (SIRI) to distinguish true tumor progression from pseudoprogression in HGG.

Accordingly, a fusion model is proposed that integrates a transformer‐derived imaging signature (Transformer‐Score) with selected clinical features—particularly systemic inflammatory markers. The model is designed to predict one‐year recurrence following multimodal therapy (surgery with adjuvant chemoradiation). Its predictions may help identify patients at risk of early recurrence and guide subsequent management—for example, consideration of radiotherapy dose escalation, enrollment in clinical trials of targeted agents, or adjustment of follow‐up frequency.

## 2 Methods and Materials

### 2.1 Patients Cohort

**FIGURE 1:**
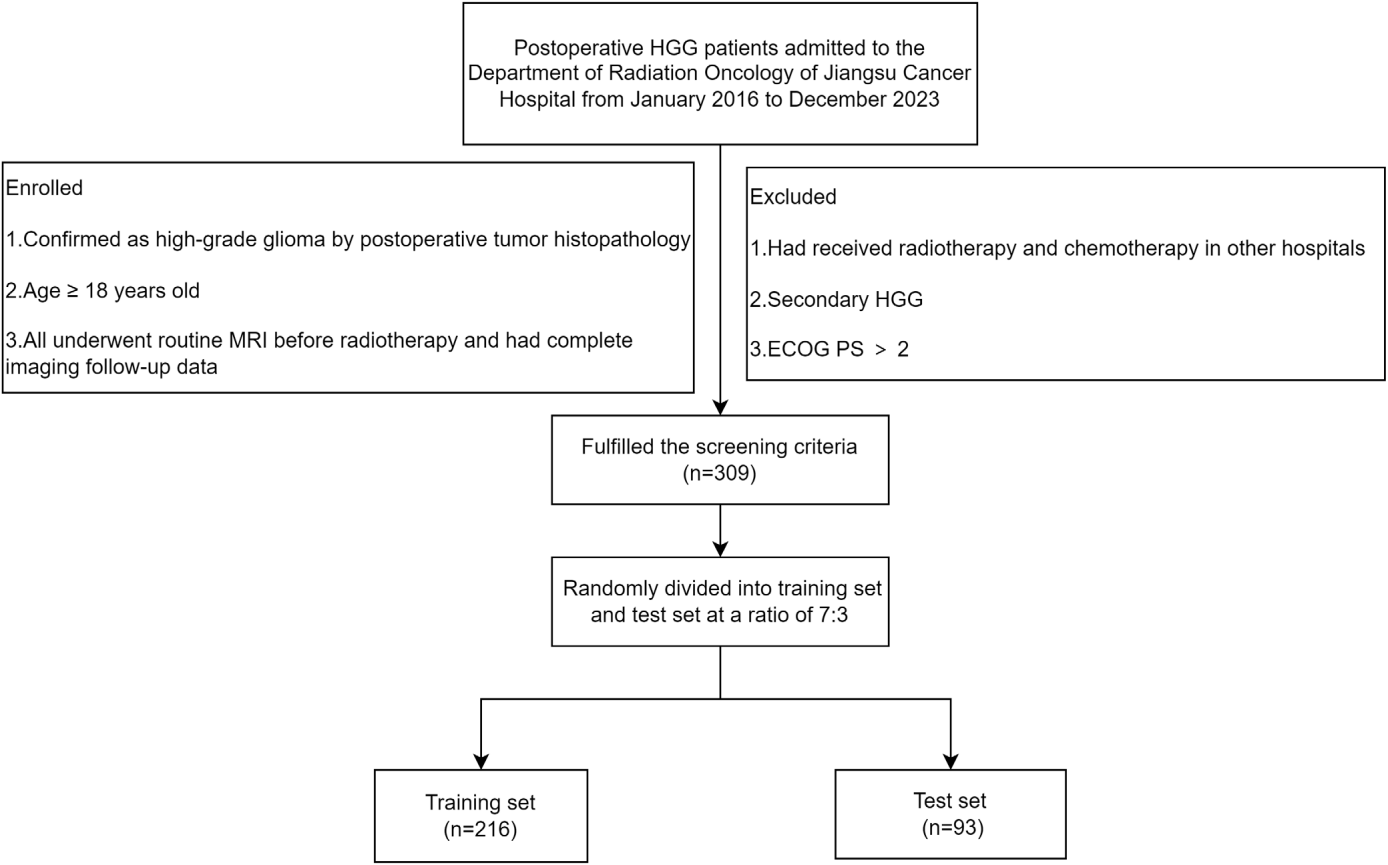
Flowchart of patient selection.

The Institutional Review Board of Jiangsu Cancer Hospital granted ethical approval (KY‐2024‐115) for the study. Due to its retrospective nature, informed consent was not required. The inclusion and exclusion criteria are summarized in Figure 1.

### 2.2 Treatment and Follow‐Up

Before radiotherapy, all patients underwent comprehensive evaluation, including physical examination, assessment of general condition, complete blood count, serum biochemistry, and a pre‐radiotherapy MRI.

Simulation CT and MRI were obtained prior to radiotherapy to generate axial CT images with 3‐mm slice thickness. Preoperative and postoperative MRI datasets were used to assist target delineation: the gross tumor volume (GTV) encompassed the contrast‐enhancing region on postoperative T1‐weighted MRI and the surgical cavity. The clinical target volume (CTV) was generated by expanding the GTV by 2 cm to include the abnormal signal on postoperative T2WI‐FLAIR, with refinement along anatomical boundaries. The planning target volume (PTV) was created by expanding the CTV by 3–5 mm. The prescription was 54–60 Gy in 30 fractions (1.8–2.0 Gy per fraction), delivered five days per week over 5–6 weeks.

Chemotherapy followed the Stupp regimen. During radiotherapy, oral temozolomide (TMZ) was administered at 75 mg/m^2^/day for 42 consecutive days. After a 4‐week interval, adjuvant TMZ was administered orally at 150–200 mg/m^2^/day on days 1–5 of each 28‐day cycle for at least six cycles.

Outpatient follow‐up was conducted after radiotherapy. Outcomes, including survival, local control, and recurrence, were ascertained through clinic re‐examinations, review of medical records, telephone contact, and WeChat communication. The last follow‐up occurred in January 2025. Brain MRI was performed every 3–4 months after radiotherapy.

Tumor recurrence or progression was assessed according to the Response Assessment in Neuro‐Oncology (RANO 2.0) imaging criteria [25] and was defined as either a ≥ 25% increase in the sum of the products of perpendicular diameters or a ≥ 40% increase in tumor volume, or the appearance of new measurable lesions.

### 2.3 Clinical Variables Collection

The following clinical variables were collected: age; body mass index (BMI); Karnofsky Performance Status (KPS); Ki‐67 index; total protein (TP); albumin (ALB); globulin (GLB); red cell distribution width (RDW); lymphocyte count (Lym); monocyte count (Mon); neutrophil count (Neu); white blood cell count (WBC); platelet count (PLT); neutrophil‐to‐lymphocyte ratio (NLR); platelet‐to‐lymphocyte ratio (PLR); lymphocyte‐to‐monocyte ratio (LMR); prognostic nutritional index (PNI); albumin‐to‐globulin ratio (AGR); systemic inflammation response index (SIRI); systemic immune‐inflammation index (SII); intervals between surgery and radiotherapy; total radiation dose; gender; comorbid hypertension or diabetes; histologic grade; pathological diagnosis; number of lesions; extent of surgical resection; IDH status; MGMT promoter methylation status; residual tumor status; fractionation scheme; and irradiation technique.

Formulas were defined as follows: PNI = albumin + 5 × lymphocyte; SIRI = (neutrophil × monocyte)/lymphocyte; SII = platelet × neutrophil/lymphocyte. Peripheral blood indices were obtained within one week prior to radiotherapy.

All patients were classified according to the fifth edition of the WHO Classification of Tumors of the Central Nervous System (WHO CNS5); therefore, grade 3 IDH‐mutant gliomas were analyzed separately and were not combined with GBM.

### 2.4 Image Preprocessing and ROI Segmentation

All MRI scans underwent N4 bias‐field correction using the SimpleITK toolkit (https://www.simpleitk.org). T1‐weighted (T1WI) and contrast‐enhanced T1‐weighted (T1CE) images were coregistered to T2‐weighted (T2WI) images using FLIRT (affine), with 12 degrees of freedom and trilinear interpolation, as implemented in the FMRIB Software Library (FSL; https://fsl.fmrib.ox.ac.uk/fsl/fslwiki/FSL). All datasets were resampled to a voxel size of 1 × 1 × 1 mm^3^ using nearest‐neighbor interpolation in SimpleITK. For 3D segmentation, ITK‐SNAP (version 3.6.0; https://www.itksnap.org) was used to delineate regions of interest on T2WI images.

The region of interest (ROI) included the postoperative cavity and peritumoral edema and was delineated independently by two senior physicians with 20 and 25 years of experience, respectively. Disagreements were resolved by consensus discussion, and the physicians were blinded to patient information. To ensure ROI consistency, 30 patients were randomly selected for interobserver reliability assessment; only features with an intraclass correlation coefficient (ICC) > 0.75 were retained for subsequent analysis.

### 2.5 Clinical Variables Selection and Model Construction

Differences in clinical features between the recurrence and stable groups were assessed using an independent‐samples *t*‐test for normally distributed continuous variables, the Mann–Whitney *U* test for non‐normally distributed continuous variables, and the chi‐square test for categorical variables. Variables associated with group status at *p* < 0.05 in univariate analyses were subsequently entered into machine‐learning algorithms—SVM, k‐nearest neighbors (KNN), and multilayer perceptron (MLP)—to develop clinical models for predicting one‐year recurrence or progression in patients with glioma.

### 2.6 Deep Learning Signature Construction

Three pretrained convolutional neural network (CNN) architectures—DenseNet‐121, DenseNet‐169, and DenseNet‐201—were used for analysis. The 3D region of interest (ROI) was delineated to encompass the postoperative cavity and peritumoral edema. Contiguous axial slices were then generated to produce 2D ROI images; the slice with the largest ROI area was selected as the representative image. Grayscale intensities were normalized to the range [−1, 1] using min–max scaling. Each ROI was cropped and resized to 224 × 224 pixels using nearest‐neighbor interpolation. The resulting preprocessed images were used as inputs to the deep‐learning models. Fivefold cross‐validation was applied to optimize hyperparameters during model training. The architectures and finalized hyperparameters are summarized in Table 1. The model with the best predictive performance was selected as the backbone for constructing the deep transfer‐learning (DTL) signature and was subsequently used to generate per‐sample probability scores.

**TABLE 1:** Architecture of the DenseNet models and optimized hyperparameter settings.

Deep neural network	Optimizer	Initial learning rate	Batch size	Epoch
DenseNet121	Sgd	0.01	32	100
DenseNet169	Sgd	0.01	32	100
DenseNet201	Sgd	0.01	32	100

*Note:* All models were trained with early stopping and 5‐fold cross‐validation on the training set.

### 2.7 Transformer‐Based Signature Construction

After model training, deep‐learning features extracted from each MRI modality were integrated using a transformer‐based approach. The transformer‐based feature‐fusion pipeline used to estimate patient prognosis proceeded as follows:

First, features were obtained by applying deep‐learning models to three MRI sequences. Second, each modality‐specific feature vector was treated as a token, analogous to a word in a sentence. Third, the tokens were passed through an embedding layer to project them into a higher‐dimensional latent space, facilitating cross‐feature interaction. Fourth, the Transformer encoder employed multi‐head self‐attention to weight tokens dynamically, enabling the model to focus on the most informative features. Subsequently, a position‐wise feed‐forward network refined the feature representations. Fifth, feature fusion: the Transformer encoder's output comprised fused representations integrating information from all three MRI sequences. Sixth, prediction: the fused representations were passed to a final classifier composed of fully connected layers. The classifier terminated in a single‐unit output with sigmoid activation, yielding the predicted probability of tumor recurrence within one year. Detailed hyperparameters of the Transformer encoder used for feature fusion are summarized in Table 2. Finally, the transformer score was treated as an independent predictor and, together with nine selected clinical variables, was entered into a multivariable logistic regression model.

**TABLE 2:** Hyperparameter configuration of transformer encoder used in feature fusion.

Model	Input feature dim	Embedding dim	No. of encoder layers	No. of attention heads	Optimizer	Initial learning rate	Positional encoding
Transformer‐DenseNet121	1024 × 3	1024	2	8	Sgd	0.01	Added (learnable)
Transformer‐DenseNet169	1664 × 3	1664	2	8	Sgd	0.01	Added (learnable)
Transformer‐DenseNet201	1920 × 3	1920	2	8	Sgd	0.01	Added (learnable)

### 2.8 Statistical Analysis

Diagnostic performance of the predictive signatures was evaluated using receiver operating characteristic (ROC) curve analysis in the training and independent validation cohorts, and model calibration was assessed with calibration plots and the Hosmer–Lemeshow goodness‐of‐fit test. To quantify clinical relevance, decision‐curve analysis (DCA) was performed for each signature to estimate net benefit across threshold probabilities. All analyses were conducted in Python 3.7.12. Statistical comparisons were performed with statsmodels (v0.13.2), including Pearson's *χ* ^2^ tests for categorical variables and independent‐samples *t*‐tests for continuous variables. Machine‐learning models included multilayer perceptrons implemented in scikit‐learn (v1.0.2) and deep neural networks developed in PyTorch (v1.11.0), with GPU acceleration (CUDA 11.3.1; cuDNN 8.2.1). Statistical significance was defined as *p* < 0.05 (two‐sided) for all hypothesis tests.

## 3 Results

### 3.1 Baseline Characteristics

**FIGURE 2:**
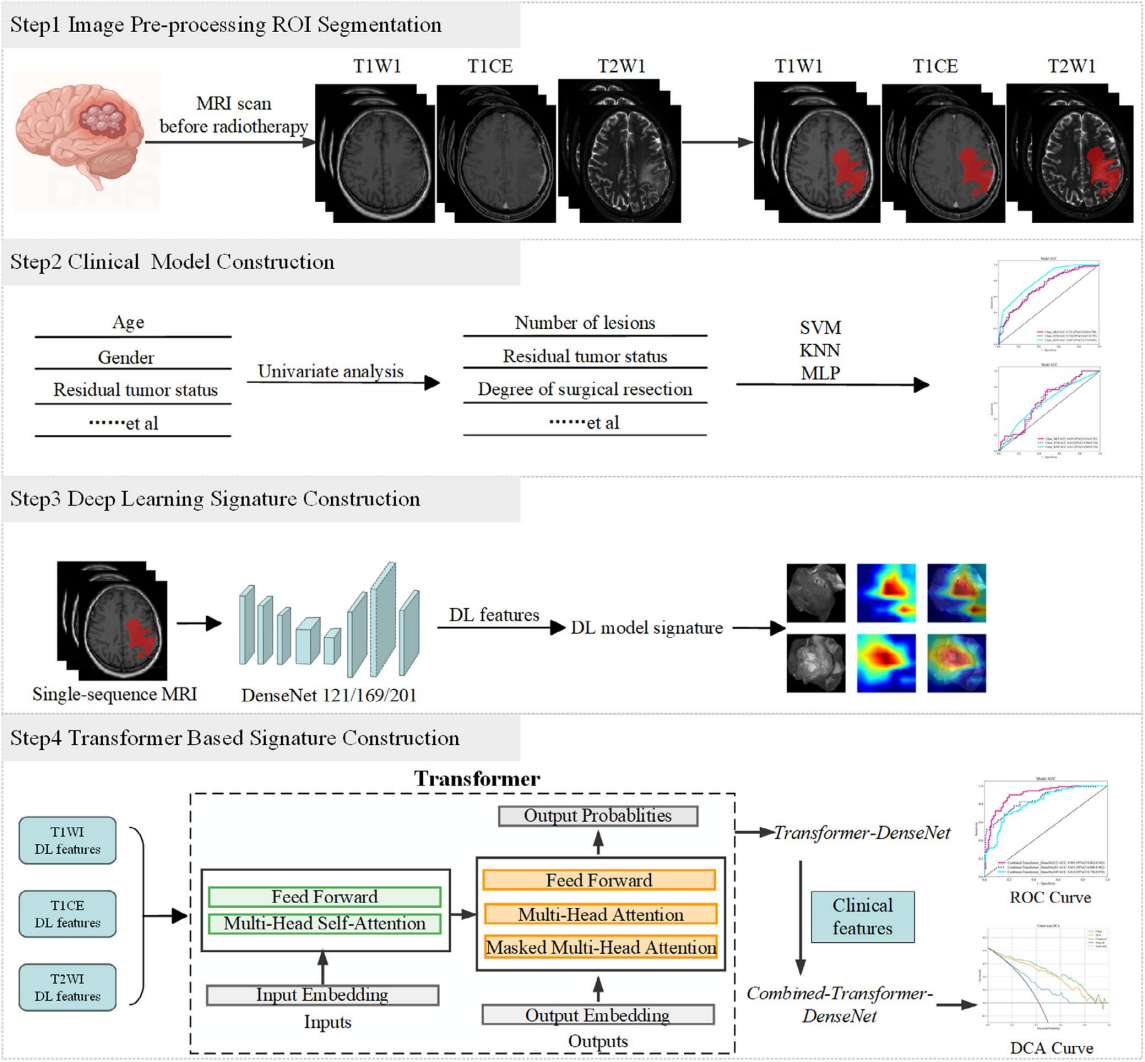
Workflow diagram of the study.

This retrospective study included 309 patients with HGG treated at Jiangsu Provincial Cancer Hospital from 2016 to 2023. Each patient was followed for at least one year through outpatient evaluations. Of the 309 patients, 134 experienced recurrence within one year. Using a 7:3 allocation ratio, patients were allocated to a training cohort (*n* = 216) and an independent test cohort (*n* = 93) for analysis (Table 3). Figure 2 depicts the overall study workflow.

**TABLE 3:** Baseline characteristics of the training and test sets.

Characteristics	Training set	Test set	*p*
	(*n* = 216)	(*n* = 93)	
Age	52.83 ± 12.52	54.48 ± 13.30	0.191
BMI	23.68 ± 3.04	23.85 ± 2.58	0.631
KPS	78.66 ± 9.08	79.78 ± 8.97	0.312
Ki‐67	29.83 ± 11.76	29.60 ± 12.97	0.690
TP (g/L)	69.96 ± 5.70	70.53 ± 5.10	0.406
ALB (g/L)	44.31 ± 4.20	44.31 ± 3.87	0.919
GLB (g/L)	25.65 ± 4.08	26.22 ± 3.89	0.184
RDW (fL)	12.30 ± 2.44	11.91 ± 2.27	0.186
Lym (10^9^/L)	1.66 ± 0.53	1.55 ± 0.48	0.178
Mon (10^9^/L)	0.48 ± 0.22	0.45 ± 0.15	0.738
Neu (10^9^/L)	4.15 ± 1.84	4.07 ± 1.82	0.886
WBC (10^9^/L)	6.44 ± 2.15	6.20 ± 2.00	0.351
PLT (10^9^/L)	251.87 ± 87.07	243.09 ± 75.46	0.464
NLR	2.70 ± 1.40	2.89 ± 1.50	0.295
PLR	163.57 ± 69.20	168.27 ± 59.54	0.278
LMR	3.95 ± 1.81	3.68 ± 1.42	0.383
PNI	52.60 ± 5.11	52.04 ± 4.70	0.365
AGR	1.77 ± 0.33	1.73 ± 0.32	0.287
SIRI	1.39 ± 1.22	1.32 ± 0.84	0.534
SII	687.92 ± 441.20	704.61 ± 437.76	0.673
Intervals between surgery and radiotherapy	35.03 ± 20.96	35.84 ± 18.26	0.759
Total radiation dose	59.88 ± 3.97	60.26 ± 3.83	0.332
Gender			
Male	133 (61.57)	57 (61.29)	1.000
Female	83 (38.43)	36 (38.71)	
Hypertension/diabetes			
Yes	57 (26.39)	37 (39.78)	0.027
No	159 (73.61)	56 (60.22)	
Histologic grade			
WHO III	45 (20.83)	13 (13.98)	0.209
WHO IV	171 (79.17)	80 (86.02)	
Pathological diagnosis			
Glioblastoma	160 (74.07)	75 (80.65)	0.273
Non‐glioblastoma	56 (25.93)	18 (19.35)	
Number of lesions			
Single	177 (81.94)	76 (81.72)	1.000
Multiple	39 (18.06)	17 (18.28)	
Degree of surgical resection			
Total removal	121 (56.02)	41 (44.09)	0.155
Partial removal	89 (41.20)	49 (52.69)	
Biopsy	6 (2.78)	3 (3.23)	
*IDH* status			
Wildtype	187 (86.57)	77 (82.80)	0.240
Mutant	29 (13.43)	16 (17.20)	
*MGMT* promoter methylation status			
Methylated	117 (54.17)	51 (54.84)	0.439
Unmethylated	99 (45.83)	42 (45.16)	
Residual tumor status			
Present	95 (43.98)	52 (55.91)	0.071
Absent	121 (56.02)	41 (44.09)	
Fractionation scheme			
Conventional fractionation	192 (88.89)	81 (87.10)	0.797
Hyperfractionation	24 (11.11)	12 (12.90)	
Irradiation technique			
IMRT	210 (97.22)	91 (97.85)	0.680
VMAT	6 (2.78)	2 (2.15)	

**FIGURE 3:**
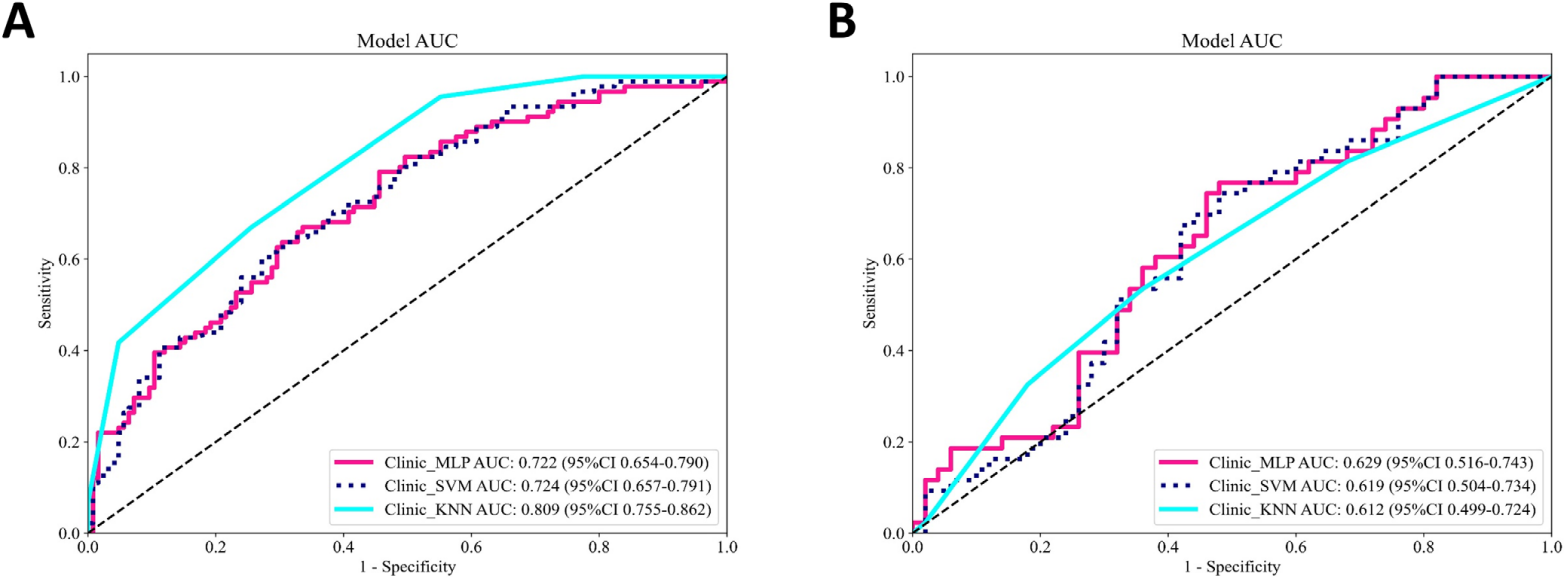
ROC curves of the machine learning model based on clinical features (A: training set; B: test set).

Univariate analyses were performed to evaluate all clinical variables. Normally distributed continuous variables were analyzed using independent‐samples *t*‐tests, whereas non‐normally distributed continuous variables were assessed with the Mann–Whitney *U* test. Categorical variables were compared using chi‐square tests to examine between‐group differences. Based on statistical significance and clinical relevance, the following variables were incorporated into the clinical and fusion models: number of lesions; residual tumor status; extent of surgical resection; pathological diagnosis; histologic grade; Mon; LMR; SIRI; and IDH mutation status (Table 4). Three machine‐learning algorithms were evaluated for clinical data integration and analysis: SVM, KNN, and MLP. Based on comparative performance using the area under the receiver operating characteristic curve (AUC), the MLP demonstrated the highest discriminative performance and was therefore selected for subsequent fusion‐model comparisons (Figure 3).

**TABLE 4:** Univariate analysis to examine differences in clinical features across groups in the training set (*n* = 216).

Clinical features	Stable group	Recurrence group	*p*
	(*n* = 125)	(*n* = 91)	
Age	51.82 ± 13.04	54.22 ± 11.70	0.189
BMI	23.64 ± 3.06	23.73 ± 3.02	0.836
KPS	79.12 ± 9.07	78.02 ± 9.09	0.348
Ki‐67	29.00 ± 12.81	30.97 ± 10.11	0.252
TP (g/L)	70.26 ± 5.65	69.55 ± 5.77	0.373
ALB (g/L)	44.77 ± 4.27	43.68 ± 4.04	0.127
GLB (g/L)	25.49 ± 3.91	25.87 ± 4.32	0.289
RDW (fL)	12.36 ± 2.54	12.23 ± 2.31	0.675
Lym (10^9^/L)	1.63 ± 0.45	1.70 ± 0.63	0.991
Mon (10^9^/L)	0.46 ± 0.24	0.51 ± 0.21	0.007
Neu (10^9^/L)	4.02 ± 1.89	4.31 ± 1.78	0.174
WBC (10^9^/L)	6.27 ± 2.14	6.69 ± 2.14	0.100
PLT (10^9^/L)	243.15 ± 89.82	263.85 ± 82.12	0.063
NLR	2.65 ± 1.46	2.77 ± 1.32	0.263
PLR	160.07 ± 74.33	168.37 ± 61.53	0.096
LMR	4.10 ± 1.75	3.76 ± 1.88	0.048
PNI	52.92 ± 5.11	52.16 ± 5.11	0.283
AGR	1.80 ± 0.33	1.74 ± 0.34	0.252
SIRI	1.31 ± 1.24	1.50 ± 1.21	0.029
SII	652.66 ± 429.35	736.36 ± 454.89	0.064
Intervals between surgery and radiotherapy	34.48 ± 24.30	35.79 ± 15.34	0.376
Total radiation dose	59.87 ± 3.89	59.89 ± 4.09	0.363
Gender			
Male	72 (57.60)	61 (67.03)	0.206
Female	53 (42.40)	30 (32.97)	
Hypertension/diabetes			
Yes	31 (24.80)	26 (28.57)	0.642
No	94 (75.20)	65 (71.43)	
Histologic grade			
WHO III	39 (31.20)	6 (6.59)	< 0.001
WHO IV	86 (68.80)	85 (93.41)	
Pathological diagnosis			
Glioblastoma	79 (63.20)	81 (89.01)	< 0.001
Non‐glioblastoma	46 (36.80)	10 (10.99)	
Number of lesions			
Single	110 (88.00)	67 (73.63)	0.011
Multiple	15 (12.00)	24 (26.37)	
Degree of surgical resection			
Total removal	82 (65.60)	39 (42.86)	< 0.001
Partial removal	37 (29.60)	52 (57.14)	
Biopsy	6 (4.80)	0	
*IDH* status			
Wildtype	100 (80.65)	87 (94.57)	0.012
Mutant	24 (19.35)	5 (5.43)	
*MGMT* promoter methylation status			
Methylated	77 (62.10)	40 (43.48)	0.083
Unmethylated	47 (37.90)	52 (56.52)	
Residual tumor status			
Present	43 (34.40)	52 (57.14)	0.001
Absent	82 (65.60)	39 (42.86)	
Fractionation scheme			
Conventional fractionation	108 (86.40)	84 (92.31)	0.252
Hyperfractionation	17 (13.60)	7 (7.69)	
Irradiation technique			
IMRT	123 (98.40)	87 (95.60)	0.057
VMAT	2 (1.60)	4 (4.40)	

### 3.2 Comparison of Different Deep Learning Models Based on Single‐Sequence MRI and Feature Extraction

Three deep‐learning (DL) models—DenseNet‐121, DenseNet‐169, and DenseNet‐201—were evaluated to assess one‐year recurrence risk. Model performance metrics, including accuracy (ACC), area under the receiver operating characteristic curve (AUC), sensitivity (SEN), specificity (SPE), positive predictive value (PPV), negative predictive value (NPV), and F1 score, are summarized in Table 5. Notably, for each MRI sequence (T1WI, T1CE, and T2WI), integrating deep‐learning features with clinical variables improved model performance, yielding higher AUCs than the corresponding single‐sequence models without clinical variables in both the training and test cohorts.

**TABLE 5:** The predictive performances of single‐sequence MRI models and their clinically integrated variants.

Cohort	Sequence	Signature	Accuracy	AUC	95% CI	Sensitivity	Specificity	PPV	NPV	F1
Train		Clinic_MLP	0.644	0.722	0.654–0.790	0.780	0.544	0.555	0.773	0.648
Train	T1	DenseNet‐121	0.657	0.669	0.595–0.743	0.451	0.808	0.631	0.669	0.526
Train	T1	Combined‐DenseNet‐121	0.750	0.807	0.750–0.865	0.747	0.752	0.687	0.803	0.716
Train	T1CE	DenseNet‐121	0.630	0.714	0.646–0.782	0.846	0.472	0.538	0.808	0.658
Train	T1CE	Combined‐DenseNet‐121	0.755	0.811	0.755–0.868	0.659	0.824	0.732	0.769	0.694
Train	T2	DenseNet‐121	0.630	0.739	0.674–0.805	0.901	0.432	0.536	0.857	0.672
Train	T2	Combined‐DenseNet‐121	0.764	0.826	0.771–0.881	0.681	0.824	0.738	0.780	0.709
Train	T1	DenseNet‐201	0.704	0.742	0.674–0.809	0.714	0.696	0.631	0.770	0.670
Train	T1	Combined‐DenseNet‐201	0.736	0.820	0.764–0.876	0.857	0.648	0.639	0.862	0.732
Train	T1CE	DenseNet‐201	0.565	0.607	0.531–0.683	0.846	0.360	0.490	0.763	0.621
Train	T1CE	Combined‐DenseNet‐201	0.685	0.771	0.709–0.833	0.890	0.536	0.583	0.870	0.704
Train	T2	DenseNet‐201	0.606	0.660	0.586–0.733	0.692	0.544	0.525	0.708	0.597
Train	T2	Combined‐DenseNet‐201	0.718	0.786	0.726–0.846	0.769	0.680	0.636	0.802	0.697
Train	T1	DenseNet‐169	0.611	0.611	0.535–0.687	0.560	0.648	0.537	0.669	0.548
Train	T1	Combined‐DenseNet‐169	0.699	0.776	0.715–0.838	0.747	0.664	0.618	0.783	0.677
Train	T1CE	DenseNet‐169	0.778	0.846	0.794–0.898	0.824	0.744	0.701	0.853	0.758
Train	T1CE	Combined‐DenseNet‐169	0.829	0.908	0.870–0.947	0.802	0.848	0.793	0.855	0.758
Train	T2	DenseNet‐169	0.653	0.634	0.558–0.710	0.385	0.848	0.648	0.654	0.758
Train	T2	Combined‐DenseNet‐169	0.722	0.777	0.716–0.839	0.681	0.752	0.667	0.764	0.758
Test		Clinic_MLP	0.624	0.629	0.516–0.743	0.744	0.520	0.571	0.703	0.646
Test	T1	DenseNet‐121	0.602	0.606	0.489–0.723	0.535	0.660	0.575	0.623	0.554
Test	T1	Combined‐DenseNet‐121	0.602	0.659	0.547–0.771	0.884	0.360	0.543	0.783	0.673
Test	T1CE	DenseNet‐121	0.624	0.655	0.543–0.768	0.558	0.680	0.600	0.642	0.578
Test	T1CE	Combined‐DenseNet‐121	0.656	0.669	0.559–0.780	0.837	0.500	0.590	0.781	0.692
Test	T2	DenseNet‐121	0.624	0.652	0.540–0.764	0.581	0.660	0.595	0.647	0.588
Test	T2	Combined‐DenseNet‐121	0.667	0.697	0.590–0.805	0.372	0.920	0.800	0.630	0.508
Test	T1	DenseNet‐201	0.645	0.635	0.518–0.753	0.605	0.680	0.619	0.667	0.612
Test	T1	Combined‐DenseNet‐201	0.645	0.667	0.556–0.778	0.488	0.780	0.656	0.639	0.560
Test	T1CE	DenseNet‐201	0.624	0.637	0.523–0.752	0.837	0.440	0.562	0.759	0.673
Test	T1CE	Combined‐DenseNet‐201	0.624	0.678	0.569–0.788	0.767	0.500	0.569	0.714	0.653
Test	T2	DenseNet‐201	0.634	0.617	0.501–0.734	0.349	0.880	0.714	0.611	0.469
Test	T2	Combined‐DenseNet‐201	0.645	0.672	0.562–0.783	0.767	0.540	0.589	0.730	0.667
Test	T1	DenseNet‐169	0.613	0.543	0.422–0.664	0.302	0.880	0.684	0.595	0.758
Test	T1	Combined‐DenseNet‐169	0.645	0.657	0.546–0.769	0.744	0.560	0.593	0.718	0.758
Test	T1CE	DenseNet‐169	0.624	0.625	0.510–0.740	0.674	0.580	0.580	0.674	0.758
Test	T1CE	Combined‐DenseNet‐169	0.634	0.659	0.547–0.771	0.860	0.440	0.569	0.786	0.758
Test	T2	DenseNet‐169	0.634	0.629	0.514–0.744	0.442	0.800	0.655	0.625	0.758
Test	T2	Combined‐DenseNet‐169	0.645	0.686	0.577–0.795	0.651	0.640	0.609	0.681	0.758

**FIGURE 4:**
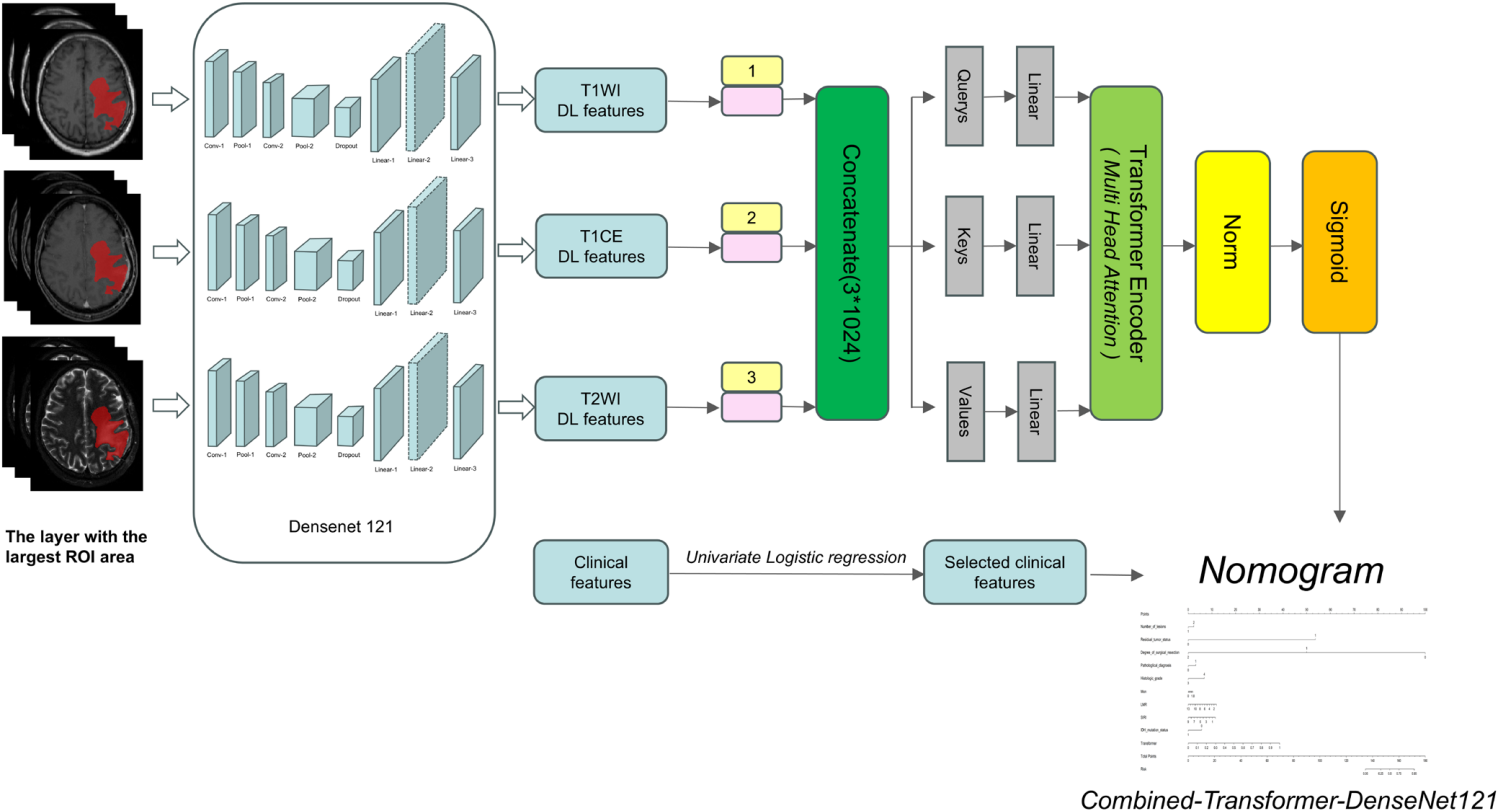
Diagram of the Combined‐Transformer‐DenseNet121 procedure.

Deep‐learning features were extracted from three architectures: DenseNet‐121 yielded 1024 features per modality, DenseNet‐201 generated 1920 per modality, and DenseNet‐169 produced 1664 per modality. These feature sets were subsequently used to develop the multimodal fusion model (Figure 4).

### 3.3 Transformer‐Based Model Construction and Assessment of Models

**FIGURE 5:**
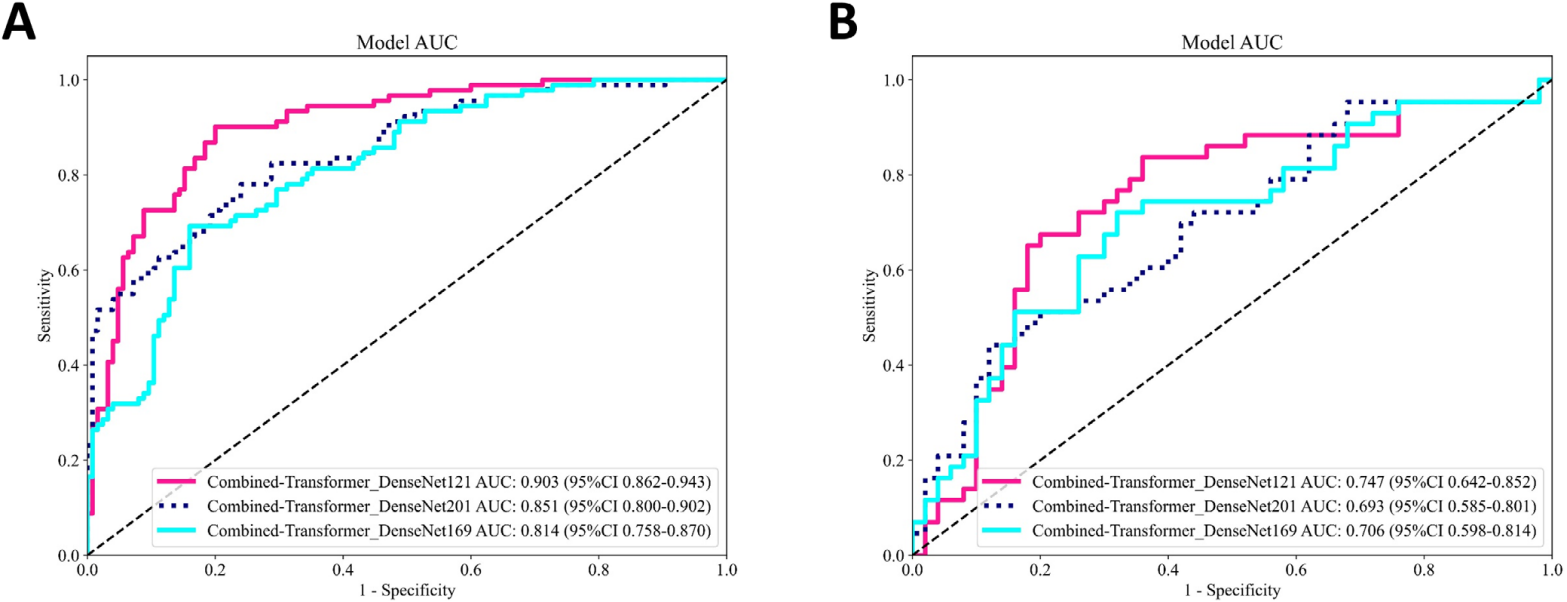
ROC curves of Combined‐Transformer‐DenseNet models (A: training set; B: test set).

Comparative analyses showed that Transformer‐enhanced fusion models consistently outperformed their single‐modality DenseNet counterparts (DenseNet‐121, DenseNet‐169, and DenseNet‐201) by AUC. Among the fusion models, Transformer‐DenseNet‐121 demonstrated the strongest performance, achieving a test AUC of 0.716, whereas Transformer‐DenseNet‐169 and Transformer‐DenseNet‐201 achieved AUCs of 0.645 and 0.693, respectively. Importantly, integrating clinical variables with Transformer‐based models yielded substantial gains (Figure 5), with training‐cohort AUCs of 0.903 (Combined‐Transformer‐DenseNet‐121), 0.851 (Combined‐Transformer‐DenseNet‐201), and 0.814 (Combined‐Transformer‐DenseNet‐169). This advantage was maintained in testing, where the enhanced Combined‐Transformer‐DenseNet‐121 achieved the highest performance (AUC = 0.747), outperforming Combined‐Transformer‐DenseNet‐169 (AUC = 0.693) and Combined‐Transformer‐DenseNet‐201 (AUC = 0.706). Comprehensive performance metrics are summarized in Table 6.

**TABLE 6:** The predictive performances of transformer‐based fusion models across training and test sets.

Cohort	Signature	Accuracy	AUC	95% CI	Sensitivity	Specificity	PPV	NPV	F1
Train	Clinic_MLP	0.644	0.722	0.654–0.790	0.780	0.544	0.555	0.773	0.648
Train	Transformer‐DenseNet121	0.796	0.871	0.821–0.921	0.879	0.736	0.708	0.893	0.784
Train	Combined‐Transformer‐DenseNet121	0.838	0.903	0.862–0.943	0.890	0.800	0.764	0.909	0.822
Train	Transformer‐DenseNet201	0.773	0.784	0.720–0.849	0.549	0.936	0.862	0.741	0.671
Train	Combined‐Transformer‐DenseNet201	0.764	0.851	0.800–0.902	0.769	0.760	0.700	0.819	0.733
Train	Transformer‐DenseNet169	0.731	0.758	0.694–0.823	0.736	0.728	0.663	0.791	0.698
Train	Combined‐Transformer‐DenseNet169	0.773	0.814	0.758–0.870	0.681	0.840	0.756	0.784	0.717
Test	Clinic_MLP	0.624	0.629	0.516–0.743	0.744	0.520	0.571	0.703	0.646
Test	Transformer‐DenseNet121	0.710	0.716	0.609–0.823	0.628	0.780	0.711	0.709	0.667
Test	Combined‐Transformer‐DenseNet121	0.720	0.747	0.642–0.852	0.814	0.640	0.660	0.800	0.729
Test	Transformer‐DenseNet201	0.667	0.645	0.528–0.761	0.372	0.920	0.800	0.630	0.508
Test	Combined‐Transformer‐DenseNet201	0.667	0.693	0.585–0.801	0.419	0.880	0.750	0.638	0.537
Test	Transformer‐DenseNet169	0.677	0.693	0.584–0.803	0.651	0.700	0.651	0.700	0.651
Test	Combined‐Transformer‐DenseNet169	0.688	0.706	0.598–0.814	0.698	0.680	0.652	0.723	0.674

**FIGURE 6:**
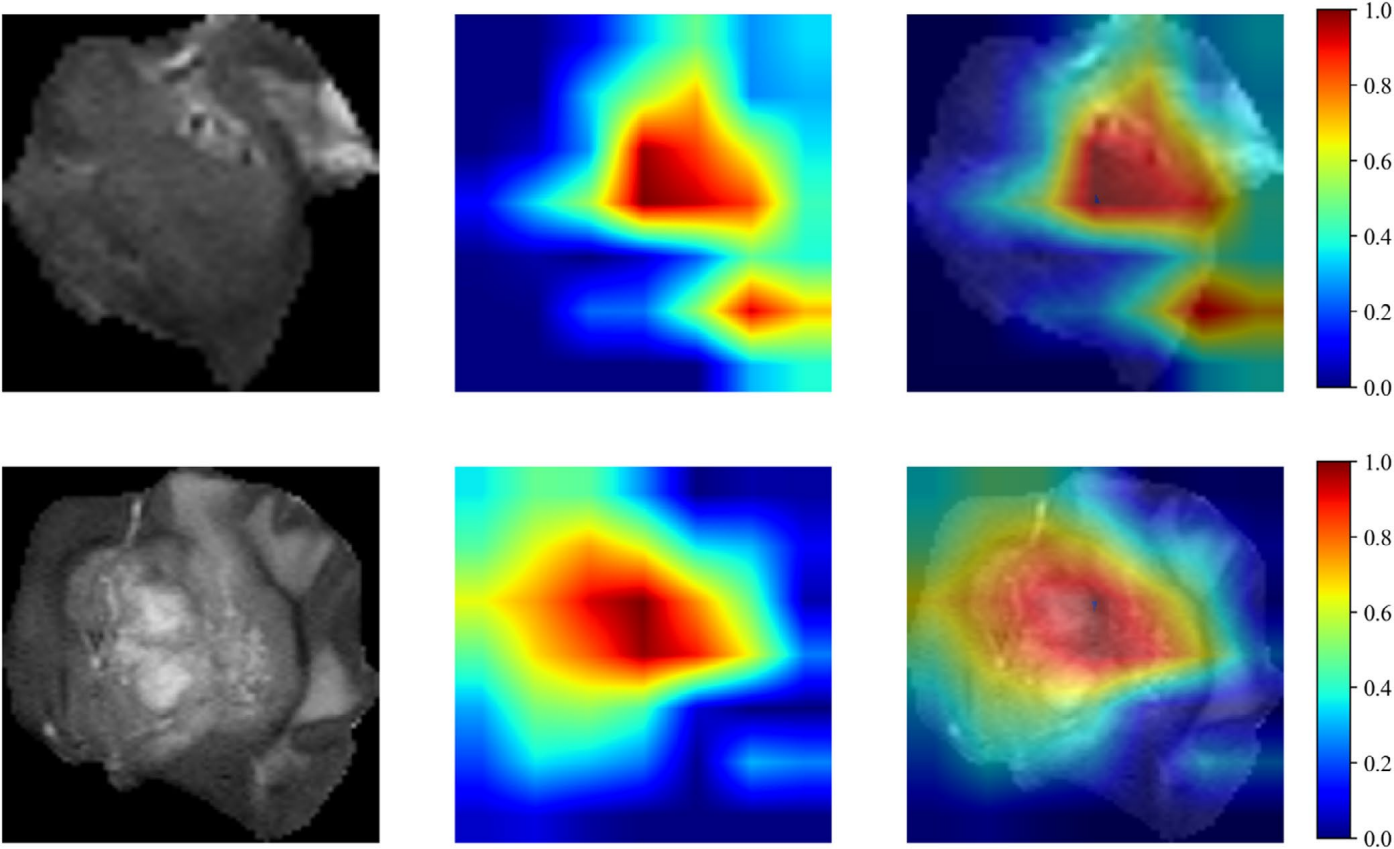
Grad‐CAM (Gradient‐weighted Class Activation Mapping) generated by DenseNet.

To illustrate model interpretability, Grad‐CAM heatmaps were generated for two patients from the test cohort. Figure 6 displays Grad‐CAM heatmaps highlighting regions to which the DenseNet assigned greater attention for the prediction of one‐year recurrence in HGG. Darker red denotes greater contribution to the prediction, whereas darker blue denotes lesser contribution.

### 3.4 Performance Metrics and Explanatory Capacity of Predictive Algorithms

**FIGURE 7:**
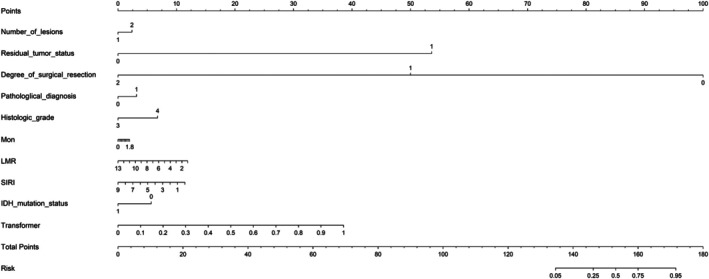
Nomogram based on the Combined‐Transformer‐DenseNet121. Number of lesions: 1: Single, 2: Multiple; Residual tumor status: 0: Absent, 1: Present; Degree of surgical resection: 0: Biopsy, 1: Partial removal, 2: Total removal; Pathological diagnosis: 0: Non‐glioblastoma, 1: Glioblastoma; Histologic grade: 3: WHO III, 4: WHO IV; IDH mutation status: 0: Wildtype, 1: Mutant.

**FIGURE 8:**
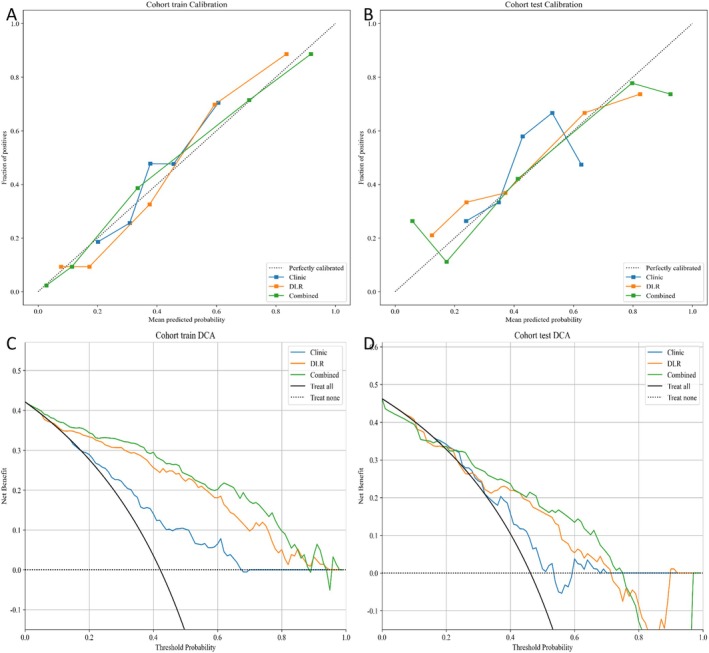
Calibration curves and DCA comparisons of the Combined‐Transformer‐DenseNet121 with other models (A, C: training set; B, D: test set).

A hybrid model was constructed by integrating the deep‐learning network with clinical predictors. As depicted by the nomogram (Figure 7), this combined approach improved calibration fidelity. Hosmer–Lemeshow tests indicated excellent agreement between predicted and observed outcomes (HL statistic, 0.166 [training] vs. 0.158 [test]), supporting robust cross‐cohort performance. DCA in both the training and test cohorts demonstrated superior clinical utility for the fusion model, which consistently yielded the highest net benefit across a range of threshold probabilities when model‐predicted probabilities were applied (Figure 8).

## 4 Discussion

HGGs are characterized by rapid growth, frequent recurrence, and poor prognosis, underscoring the clinical value of aggressive, individualized treatment. In recent years, several studies [26, 27] have applied ^11^C‐methionine positron emission tomography (^11^C‐MET PET) to HGGs for target volume delineation, radiotherapy optimization, and assessment of tumor burden or recurrence patterns. Similarly, 18F‐FDOPA PET has shown high diagnostic accuracy in GBM, improving target volume definition and facilitating the differentiation between true tumor recurrence and treatment‐related changes such as radiation necrosis [28].

This study aimed to predict one‐year recurrence in patients with HGG by integrating accessible clinical variables (treatment data, molecular pathology results, and hematologic parameters) with deep‐learning features. Such a comprehensive evaluation may facilitate identification of high‐risk patients, supporting recommendations to adjust follow‐up strategies or to encourage enrollment in feasible and effective clinical trials for this subgroup.

The deep‐learning model was based on DenseNet‐121, a convolutional neural network (CNN) architecture. The core innovation of DenseNet is its dense‐connectivity mechanism: within each dense block, every layer receives the feature maps of all preceding layers and passes its own feature maps to all subsequent layers. This mechanism promotes gradient flow, alleviates the vanishing‐gradient problem in deep networks, and improves parameter efficiency [29].

However, DenseNet is primarily adept at extracting local, hierarchical convolutional features. To better model global context and long‐range dependencies, a Transformer module was integrated, building on the rich hierarchical features extracted by DenseNet. Specifically, a Transformer‐based attention module was embedded within the DenseNet architecture. Through self‐attention, the Transformer dynamically captures complex spatial dependencies among elements within feature maps [30], thereby enhancing the model's understanding of global image semantics.

To our knowledge, this is the first study to apply deep learning to multimodal pre‐radiotherapy MRI for one‐year recurrence prediction in HGG. Pre‐radiotherapy MRI was combined with clinical variables to demonstrate its value for identifying one‐year recurrence. In the final model (Combined‐Transformer‐DenseNet‐121), a multivariable logistic regression was used to integrate the Transformer‐Score with selected clinical variables, and a nomogram was constructed from this joint model.

In addition, the potential predictive value of systemic inflammatory markers, particularly ratio‐based indices (e.g., LMR, SIRI), is highlighted; these indices integrate multiple hematologic parameters and are noninvasive, cost‐effective, and sensitive for predicting recurrence and progression.

This study has several limitations. First, critical molecular pathology variables with prognostic significance, such as 1p/19q codeletion status [31] and TERT‐promoter mutations [32], were excluded because of excessive missingness, which may have led to under‐identification of high‐risk patients (increased false‐negative rates). Second, the workflow involved manual steps: two senior physicians identified and delineated the ROIs, and future implementation will therefore require AI‐assisted automatic or semi‐automatic 3D segmentation to improve consistency and scalability. Third, the model was developed and tested in a single‐center cohort of limited size, which may introduce selection bias and restrict the generalizability of the results; the modest performance in the independent test cohort (AUC, 0.747; 95% CI, 0.642–0.852) suggests a degree of overfitting and limited external validity. Consequently, future work will focus on integrating AI‐assisted automatic or semi‐automatic 3D segmentation, performing external validation in larger, multicenter cohorts, and incorporating additional MRI modalities (e.g., functional sequences) to improve the robustness and clinical utility of the model.

## 5 Conclusion

In summary, a transformer‐based fusion model is presented for predicting one‐year posttreatment recurrence in HGG. Substantial improvements in the efficiency and accuracy of recurrence or progression risk stratification were observed.

## Author Contributions

**Xin Wang:** conceptualization, formal analysis, investigation, data curation, writing – original draft, visualization. **Mingjun Ding:** methodology, validation, writing – original draft, visualization. **Dan Zong:** writing – review and editing. **Pudong Qian:** writing – review and editing. **Xia He:** supervision.

## Funding

This paper was supported by National Natural Science Foundation of China (No. 82172804), Key Project of Jiangsu Provincial Health Commission (No. K2019028), Nanjing Science and Technology Plan Project (No. 2022SX00001663).

## Ethics Statement

This study was conducted in accordance with the Declaration of Helsinki and was approved by the Ethics Review Committee of The Affiliated Cancer Hospital of Nanjing Medical University with an approval number of KY‐2024‐115.

## Conflicts of Interest

The authors declare no conflicts of interest.

## Data Availability

Data is provided within the manuscript; further inquiries can be directed to the corresponding authors.
